# Accuracy of blood-pressure monitors owned by patients with hypertension (ACCU-RATE study): a cross-sectional, observational study in central England

**DOI:** 10.3399/bjgp20X710381

**Published:** 2020-06-02

**Authors:** James A Hodgkinson, Mei-Man Lee, Siobhan Milner, Peter Bradburn, Richard Stevens, FD Richard Hobbs, Constantinos Koshiaris, Sabrina Grant, Jonathan Mant, Richard J McManus

**Affiliations:** Institute of Applied Health Research, University of Birmingham, Edgbaston.; Nuffield professor of primary care health sciences;; Institute of Applied Health Research, University of Birmingham, Edgbaston.; Institute of Applied Health Research, University of Birmingham, Edgbaston.; Nuffield professor of primary care health sciences;; Nuffield professor of primary care health sciences;; Nuffield Department of Primary Care Health Sciences, University of Oxford, Oxford.; Three Counties School of Nursing and Midwifery, University of Worcester, Worcester.; Primary Care Unit, Strangeways Research Laboratory, Cambridge.; Nuffield Department of Primary Care Health Sciences, University of Oxford, Oxford.

**Keywords:** accuracy, blood-pressure monitors, calibration, hypertension, primary health care

## Abstract

**Background:**

Home blood-pressure (BP) monitoring is recommended in guidelines and is increasingly popular with patients and health professionals, but the accuracy of patients’ own monitors in real-world use is not known.

**Aim:**

To assess the accuracy of home BP monitors used by people with hypertension, and to investigate factors affecting accuracy.

**Design and setting:**

Cross-sectional, observational study in urban and suburban settings in central England.

**Method:**

Patients (*n* = 6891) on the hypertension register at seven practices in the West Midlands, England, were surveyed to ascertain whether they owned a BP monitor and wanted it tested. Monitor accuracy was compared with a calibrated reference device at 50 mmHg intervals between 0–280/300 mmHg (static pressure test); a difference from the reference monitor of +/−3 mmHg at any interval was considered a failure. Cuff performance was also assessed. Results were analysed by frequency of use, length of time in service, make and model, monitor validation status, purchase price, and any previous testing.

**Results:**

In total, 251 (76%, 95% confidence interval [95% CI] = 71 to 80%) of 331 tested devices passed all tests (monitors and cuffs), and 86% (CI] = 82 to 90%) passed the static pressure test; deficiencies were, primarily, because of monitors overestimating BP. A total of 40% of testable monitors were not validated. The pass rate on the static pressure test was greater in validated monitors (96%, 95% CI = 94 to 98%) versus unvalidated monitors (64%, 95% CI = 58 to 69%), those retailing for >£10 (90%, 95% CI = 86 to 94%), those retailing for ≤£10 (66%, 95% CI = 51 to 80%), those in use for ≤4 years (95%, 95% CI = 91 to 98%), and those in use for >4 years (74%, 95% CI = 67 to 82%). All in all, 12% of cuffs failed.

**Conclusion:**

Patients’ own BP monitor failure rate was similar to that demonstrated in studies performed in professional settings, although cuff failure was more frequent. Clinicians can be confident of the accuracy of patients’ own BP monitors if the devices are validated and ≤4 years old.

## INTRODUCTION

Raised blood pressure (BP) is a key risk factor for the development of cardiovascular disease,^1^ a major cause of morbidity and mortality worldwide.^2^ An accurate BP monitoring device is fundamental to the diagnosis and management of hypertension.

Self-monitored BP at home is a statistically significantly better predictor of future cardiovascular risk than manual office BP measurement,^3^ and self-monitoring as part of a self-management strategy is an effective way to improve BP control.^4^^,^^5^ Home BP monitoring has gained popularity in recent years among both patients and healthcare professionals (HCPs), many of whom incorporate self-monitored readings in their treatment decisions;^6^ nevertheless, there is considerable variation in practice, and there remains scepticism among some HCPs about the accuracy of patients’ own readings, especially outside of a trial context.^6^^,^^7^

Although guidance on how to conduct self-monitoring of BP recommends use of validated upper-arm cuff devices, appropriate training, use of a pre-specified schedule (for example, number of days of readings, time of day), and physician verification of measurements,^8^^,^^9^ none to date recommends checking the accuracy of home BP monitors used by patients. Previous research has highlighted that monitors used in GP surgeries and community pharmacies have shown variation in accuracy.^10^^,^^11^

Several clinical protocols^12^^–^^15^ exist for the validation of BP measuring devices but these are, generally, undertaken on brand-new models and do not assess sustained accuracy thereafter. Typically, new monitors are assumed to be accurate for 2 years and then annual checks are undertaken in clinical practice. However, it is not clear whether this is appropriate as the drift in accuracy, over time, of an automated sphygmomanometer is not known, and a study investigating monitors in pharmacies suggested they decline in accuracy after 18 months.^11^

Some automated BP monitors on sale to the public have been clinically validated; in such cases, a monitor or one with device equivalence^16^ will have passed at least one of the recognised accuracy protocols.^12^^–^^14^ However, error rates in devices used for self-monitoring are unknown; this rate is a function of random error (variability) and systematic error (bias), and, ultimately, depends on the conditions under which a device is used.

This study aimed to test — for the first time in the UK, to the authors’ knowledge — the accuracy of monitors in use by the general public for the self-monitoring of BP. Secondary aims included to:

- determine which automated sphygmomanometers were currently used by patients;
- assess factors affecting accuracy, including those makes and models that performed best; and
- evaluate the influence of regular use and length of time in service on accuracy.

**Table:** How this fits in

Self-monitoring blood pressure (BP) is common, but the accuracy of patients’ own monitors is currently unclear. This study provides evidence that the accuracy of some monitors used at home is similar to that of those used in professional settings, albeit with more frequent cuff failure. The study also found that validated monitors, those costing >£10, and those in use for ≤4 years were more likely to perform better. Clinicians can be reassured that patients’ own BP monitors are likely to be accurate if a validated model that is ≤4 years old is being used.

## METHOD

Patients on the hypertension register at seven practices in the West Midlands (in central England), UK, were sent an invitation letter together with a one-page questionnaire and a self-addressed envelope. The questionnaire (Supplementary Information S1) asked if they owned a BP monitor and, if so, some basic questions about it and whether they wanted its accuracy to be assessed free of charge — this required them to bring the monitor into the practice at a prearranged time when they would meet a member of the research team. Practices were purposefully sampled by social deprivation (based on Index of Multiple Deprivation [IMD] 2010 scores) in order to achieve a diverse sample of monitors that were likely to range in affordability. IMD scores varied from 6.09 to 49.58.

Testing took place between March 2016 and August 2017. Following visual inspection (that the machine switched on and had a readable display), the accuracy of each testable digital sphygmomanometer was evaluated by comparing it with a calibrated reference digital BP monitor tester (Omron PA350); tests were conducted at 50 mmHg intervals across a range of 0–300 mmHg following a standard process, as recommended by each monitor manufacturer and the British Hypertension Society.^12^ A difference from the reference monitor of +/−3 mmHg at any testing interval was considered a failure. In addition to this static pressure test, monitors and cuffs underwent fast deflation tests (pass threshold: deflation from 260 mmHg to 15 mmHg in <10 seconds) and air-leakage checks (pass threshold: loss of <6 mmHg over 60 seconds at a stabilised pressure of 280 mmHg). Results were documented on a monitor testing form (Supplementary Information S2). With a conservative assumption of a failure rate of 50%, it was estimated that a sample size of 385 was required, using a 95% confidence interval (CI) width of +/−5%.

The mean absolute error (MAE) for each monitor was calculated as the average (arithmetic mean) of the absolute difference — whichever is larger and positive of monitor blood pressure minus reference blood pressure, and reference blood pressure minus monitor blood pressure — at each test. The relationship between monitor accuracy and make and model, length of time in use, frequency of recorded uses, monitor purchase price, and validation status was assessed using linear regression with MAE as the outcome. All model assumptions were checked. Failure rate by the different predictors was assessed using Fisher’s exact test statistic.

## RESULTS

### Sample

In total, 6891 patients appearing on the hypertension register for seven GP practices were invited to take part; 1543 (22%) responses were received. Of these, 653 (42%) patients owned monitors, of whom 526 (81%) expressed an interest in having their monitor tested. A total of 410 (78%) of the 526 monitors were provided for testing; 79 (19%) of these proved untestable because of the impossibility of separating the monitor from the cuff to test each component independently — these were, typically, wrist monitors. The 331 monitors tested comprised more than 50 different models, with the majority coming from three manufacturers: Boots (*n* = 62), Lloyds (*n* = 131), and Omron (*n* = 108).

### Device accuracy

In total, 250 devices (76%, 95% CI = 71 to 80%) passed on all tests (monitors and cuffs); 49 (15%, 95% CI = 11 to 18%) monitors failed, largely on the accuracy test (*n* = 46; 14%, 95% CI = 10 to 18%), and 39 (12%, 95% CI = 8 to 15%) overestimated pressure. Table 1 details the MAE between the reference and tested devices. Four monitors had internal corrosion or could not hold pressure and, as such, could not be subjected to the full range of testing once they had definitively failed.

**Table 1.** Mean absolute error between the values reported by the reference device and test devices (*n* = 327) at the pressure intervals tested^a^

**Test pressure, mmHg^b^**	**MAE, mmHg (95% CI)**	**Failing monitors, *n* (%)**	**Overestimation by ≥3 mmHg, *n***	**Underestimation by ≥3 mmHg, *n***
0	0.27 (0.23 to 0.30)	0 (0)	0	0
50	0.59 (0.54 to 0.65)	3 (1)	1	2
100	0.78 (0.70 to 0.86)	6 (2)	4	2
150	0.98 (0.88 to 1.09)	23 (7)	21	2
200	1.14 (1.01 to 1.27)	30 (9)	28	2
250	1.34 (1.18 to 1.49)	41 (13)	35	6
Maximum	1.46 (1.29 to 1.63)	43 (13)	37	6

a Four monitors could not be tested fully but should be counted as failures.

b Pressure is checked in 50 mmHg increments up to either 280 mmHg or 300 mmHg (according to manufacturer’s specification, the highest pressure point monitors can be tested at is either 280 mmHg or 300 mmHg) and then down again. The data at each testing interval between 0 mmHg and 250 mmHg going up have been combined with that going down. CI = confidence interval. MAE = mean absolute error.

The largest difference from the reference monitor was 11.4 mmHg (data not shown). In total, 17 (5% (95% CI = 3 to 8%) monitors failed by >5 mmHg (data not shown) and 23 (7%, 95% CI = 4 to 10%) failed at the 150 mmHg level, which is closest to the threshold used for diagnosis and treatment. The overall MAE (all monitors tested) rose at each tested pressure interval to 1.5 mmHg (95% CI = 1.3 to 1.6 mmHg) at 280/300 mmHg (depending on the maximum specified pressure for a given monitor), compared with 0.6 mmHg (95% CI = 0.5 to 0.7 mmHg) at 50 mmHg and 1.0 mmHg (95% CI = 0.9 to 1.1 mmHg) at 150 mmHg (Table 1).

### Length of time in service

Table 2 details the length of time in service of the monitors tested. In total, there was no information on years in service for 48 monitors. Of those monitors on which the full range of tests were performed and for which owners could provide a reasonable estimate regarding the number of years in service (*n* = 279), 188 (67%) had been in use for >2 years, and some for substantially longer — 61 (22%) monitors had been in use for >7 years and one was reported as having been in use for >20 years. Overall, the MAE tended to increase with length of time in service (*P*<0.001), though sample sizes were small in some categories (such as 6–7 years and >10 years). The failure rate was 5% (8/155) for the first 4 years in service, rising to 26% (32/124) for older models.

**Table 2.** Mean absolute error compared with reference device, and failure rate of tested monitors (*n* = 327) by length of time in service^a^

**Years in hservice**	**Monitors, *n***	**Failing monitors, *n* (%)^b^**	**Cumulative failing monitors, *n* (%)^c^**	**MAE, mmHg (95% CI)**
0–1	41	0	0	0.63 (0.55 to 0.72)
>1–2	50	5 (10)	5 (5)	0.73 (0.66 to 0.81)
>2–3	36	1 (3)	6 (5)	0.62 (0.53 to 0.71)
>3–4	28	2 (7)	8 (5)	0.80 (0.70 to 0.90)
>4–5	29	6 (21)	14 (8)	1.03 (0.93 to 1.13)
>5–6	20	7 (35)	21 (10)	1.44 (1.32 to 1.56)
>6–7	14	2 (14)	23 (11)	0.88 (0.73 to 1.02)
>7–10	54	15 (28)	38 (14)	1.18 (1.10 to 1.25)
>10	7	2 (29)	40 (14)	1.29 (1.08 to 1.50)
Not stated/monitor owner unable to remember	48	6 (13)	n/a	0.89 (0.81 to 0.97)

a Four monitors could not be tested fully but should be counted as failures.

b Percentage of total monitors per years-in-service classification.

c Percentage of cumulative total of monitors per years-in-service classification and shorter lengths of service. MAE = mean absolute error.

### Previous testing

Only 58 (9%) of the initial 653 responders reported having had their device tested previously: 22 said their monitors had been tested within the previous 2 years, 25 said they had been tested ≥2 years ago, and 11 gave no details about the date of previous testing (data not shown). Of the 58 responders, 40 checked device accuracy by comparing results with readings generated by an HCP (GP/nurse/pharmacist) and five had checked their machine with the manufacturer; the remaining 13 provided no information. Of 26 previously tested monitors tested again by the authors, eight failed (31%, 95% CI = 26 to 36%, *P* = 0.48 for difference between previously tested and never tested).

### Frequency of use

Table 3 shows the estimates regarding how often the devices were used. There appeared to be only a limited relationship between the frequency of use and the MAE. The failure rate for monitors used once a month or more was 9% (17/183) compared with 22% (28/129) for those used less than once a month (*P*<0.01).

**Table 3.** Mean absolute error compared with reference device and failure rate of monitors (*n* = 327) by frequency of use^a^

**Frequency of use**	**Monitors, *n***	**Failing monitors, *n* (%)^b^**	**MAE, mmHg (95% CI)**
>10 times a month	27	3 (11)	0.89 (0.84 to 0.94)
6–10 times a month	41	2 (5)	0.74 (0.69 to 0.78)
1–5 times a month	115	12 (10)	0.86 (0.84 to 0.89)
6–11 times a year	57	13 (23)	1.08 (1.04 to 1.11)
1–5 times a year	44	10 (23)	0.96 (0.92 to 1.00)
Less frequently	28	5 (18)	0.94 (0.88 to 0.99)
Not stated	15	1 (7)	0.67 (0.60 to 0.74)

a Four monitors could not be tested fully but should be counted as failures.

b Percentage of total monitors per frequency-of-use classification. CI = confidence interval. MAE = mean absolute error.

### Validation

Of those monitor models for which the validation status could be definitively identified (*n* = 317), 218 (69%) were validated and 99 (31%) were not: 209 (96%, 95% CI = 94 to 98%) of the validated monitors passed all the device tests compared with 63 (64%, 95% CI = 58 to 69%) of the unvalidated monitors (*P*<0.001 for the comparison).

### Cuff functionality

Table 4 shows that 287 (78%, 95% CI = 74 to 82%) of the cuffs were medium-sized (22–32 cm) and 57 (15%) large; it should be noted that some devices had multiple cuffs. Cuff air leakage resulted in failure for 44 (12%) of 369 cuffs (95% CI = 8 to 15%). The failure rate was higher (*P* = 0.002) in large cuffs (26%, 95% CI = 22 to 31%) than in those that were medium sized (10%, 95% CI = 7 to 13%). Other cuff-size categories had too few cases to be evaluated. Failure of the cuff air-leakage tests contributed to the overall failure rate as described above.

**Table 4.** Failure rates of cuffs by cuff size

**Cuff size (cm)**	**Cuffs tested, *n*^a^**	**Failures, *n* (%)^b^**	**95% CI**
Small (17–22)	2	0 (0)	n/a
Medium (22–32)	287	28 (10)	7 to 13
Large (32–42)	57	15 (26)	22 to 31
Extra large (42–48)	1	0 (0)	n/a
Universal — medium–large (22–42)	19	0 (0)	n/a
Other/unclear	3	1 (33)	n/a

a Some monitors came with multiple cuffs.

b Percentage of total cuffs per size classification.

### Purchase price

The reported original purchase price of devices varied from £5 to just over £100, with one outlier costing £240 and another acquired for free. Table 5 shows the relationship between purchase price and failure rate for those devices with data for both variables (*n* = 240). The vast majority (188/240, 78%) cost ≤£30, with the modal decile being £11–20 (*n* = 100). Monitor failure rate was highest for the cheapest machines (14 [34%] of 41 devices costing £1–10 failed); it improved as devices became more expensive, and (3 [6%] of the 52 devices costing >£31 failed [*P*<0.001]). However, including cuff failure rate resulted in no difference overall in failure by device cost.

**Table 5.** Failure rate by approximate purchase price of monitor

**Purchase price, £**	**Pass**	**Monitor pass, cuff fail, *n***	**All monitor pass, *n* (%)^a^**	**Monitor fail, *n* (%^a^)**	**All fail, *n* (%)**	**Total, *n***
0–10	27	0	27 (66)	14 (34)	14 (34)	41
11–20	77	10	87 (87)	13 (13)	23 (23)	100
21–30	40	3	43 (91)	4 (9)	7 (15)	47
>31	41	8	49 (94)	3 (6)	11 (21)	52
**Total**	**185**	**21**	**206**	**34**	**55**	**240**
*Full breakdown for monitors costing >£30*						
31–40	13	1	14	1	2	15
41–50	8	3	11	1	4	12
51–60	8	2	10	0	2	10
61–70	2	0	2	0	0	2
71–80	3	2	5	1	3	6
81–90	1	0	1	0	0	1
91–100	5	0	5	0	0	5
>100	1	0	1	0	0	1

a Percentage of total monitors by purchase-price classification.

### Regression analysis

A regression model identified that length of time in service (8% increase in MAE for each additional year of service) and validation status (validated models having a 23% decrease in MAE compared with unvalidated monitors) were statistically significant predictors of MAE, but estimated frequency of use, previous testing, and cost of device were not (Table 6).

**Table 6.** Regression model — mean absolute error^a^

	**Estimate**	**95% CI**	***P*-value**
**Intercept**	0.62	0.37 to 1.04	0.051

**Length of time in service**			
**Years in service (each additional year)**	1.08	1.04 to 1.12	<0.001

**Frequency of use**			
>10 times per month	0.94	0.66 to 1.38	0.76
6–10 times per month	0.82	0.61 to 1.12	0.19
1–5 times per month	1	reference	
6–11 times per year	0.90	0.68 to 1.20	0.47
1–5 times per year	0.94	0.66 to 1.36	0.74
Less frequently	0.89	0.61 to 1.32	0.53
Not known	0.80	0.38 to 2.07	0.61

**Cost of device**			
Cost (every £1 increase)	1.00	0.99 to 1.01	0.75

**Tested (reference: no)**			
Yes	1.21	0.80 to 1.91	0.38
Not known	1.10	0.87 to 1.41	0.44

**Make (reference: Boots)**			
Lloyds	1.31	0.90 to 1.90	0.13
Omron	1.01	0.75 to 1.37	0.94
Other	1.23	0.80 to 1.92	0.34

**Validation status (reference: not validated/not applicable)**			
Validated	0.77	0.59 to 1.02	0.044

a The proportion in MAE change is the unit of predictor unless otherwise stated. MAE = mean absolute error.

Due to the sheer diversity of models encountered, the intention to conduct an analysis of the performance of different makes and models proved impossible. In the regression model, any discernible difference in performance characteristics was explained by the validation status of the device type.

## DISCUSSION

### Summary

This first study (to the authors’ knowledge) of accuracy of patients’ own monitors in the UK found that approximately three-quarters of monitors and/or the matching cuff passed a standard calibration test. Inaccurate monitors generally overestimated BP, and large cuffs were more than twice as likely to fail as those that were medium sized. Validated monitors, those costing >£10, and those that were ≤4 years old were most likely to be accurate.

### Strengths and limitations

This work provides robust data to answer a question often raised by clinicians^6^ —namely, ‘how accurate is patients’ own BP monitoring equipment?’ — and one that is important in terms of planning the implementation of BP self-monitoring on a wider scale. Assessing a large number of monitors across several practices covering different sociodemographic strata provides reassurance that these results are likely to be generalisable, more so than previous smaller studies, despite a response rate of <25%. Fewer than one in 10 monitors had had any kind of previous evaluation, so such information is important.

It should be noted, however, that it was only possible to assess the accuracy of monitors that participants brought to be tested, which may represent a biased sample. A number of monitor types — primarily, wrist monitors for which there is no way of separating the cuff from the monitor — could not be tested using the researchers’ standard calibration equipment (Omron PA350); however, current guidance recommends the use of upper-arm devices, which the authors were, in general, able to test.

Data on frequency of use, length of time in service, purchase price, and previous testing were reliant on participant recall and, as such, may be subject to confounding, for example, devices in which users have more confidence (because of their apparent accuracy) may be used more frequently. However, any kind of evaluation of potential factors explaining variations in monitor performance is, to the authors’ knowledge, unique to this study.

### Comparison with existing literature

At 42%, ownership of home BP monitors in the study presented here was slightly higher than in previous published surveys of patients with hypertension in the UK,^17^ but is in keeping with GPs’ estimates of patient self-monitoring.^6^ This is perhaps unsurprising, given the likelihood of preferential responses from monitor owners who wanted their equipment tested, although the authors emphasised also being interested in receiving null responses and included a self-addressed envelope to encourage all those contacted to respond.

Previous work from outside of the UK has generally found much worse performance than found in the study presented here: a Canadian study^18^ conducted between 2011 and 2014 found that around a third of patients’ monitors showed a difference of >5 mmHg (systolic and/or diastolic) compared with a mercury measurement. No statistically significant difference was found between monitors that were accurate versus those that were not when grouped according to patient characteristics, cuff size, or the brand of the home monitor in the Canadian study.^18^ Even greater inaccuracy was identified by a different Canadian group, with 69% of devices showing differences of ≥5 mmHg and no improvement in performance for validated machines.^19^ However, a Korean study^20^ using the same methodology found monitor failure rates of 15% — similar to those in the study presented here — and that inaccuracy was more common in unvalidated devices (19%, 25/130) than those that were validated (7%, 6/82).

A Turkish study,^21^ again using similar methods (although with 4 mmHg as the threshold for failure), identified inaccuracy rates of 59%, inaccuracy rates of 67% in 119 upper-arm devices. The same sample of monitors showed accuracy was statistically significantly greater in validated devices (*n* = 22) compared with unvalidated devices (*n* = 52) (68% versus 15%, *P*<0.01).^22^ Conversely, an earlier Canadian study^23^ found no difference in monitor performance dependent on validation status but, again, included very few monitors (*n* = 26) that had been validated. The research presented here confirms the importance of using validated devices, which is generally called for in guidelines.^9^

Although it is a concern that several wrist monitors were not assessable and almost a quarter of the equipment (including cuffs) failed, the overall monitor failure rate of 15% is similar to that previously identified in devices used in general practice (13%)^10^ and pharmacies (14%).^11^ In those settings, devices were used more frequently but for shorter periods. Given that the authors of the study presented here employed quite stringent criteria — with a difference of 3 mmHg throughout the range being enough to constitute failure — this suggests the majority of home BP monitors can be considered reliable enough for use in primary care, especially those that are newer and validated.

### Implications for practice

An accurate BP monitor is fundamental to the diagnosis and management of hypertension. Self-monitoring BP devices are currently not prescribed on the NHS and, to be able to recommend home monitoring of BP more widely, there needs to be confidence in the devices accessible to patients or an ability to provide clear guidance on which models to trust, and how long for. Monitor manufacturers typically recommend annual calibration after 2 years’ service. The fact that a small proportion of home monitors in use appear to be very inaccurate does suggest the need for regular performance checks, although a more pragmatic approach might be to restrict this to unvalidated monitors or validated models that are >4 years old.

This study suggests that validation status is a reasonable indicator of both short- and longer-term performance; HCPs should be encouraged to provide patients with clear advice on this. Given the issues with cuff failure noted in this study, it might be beneficial for manufacturers to develop quality-control algorithms that alert users when cuffs are not performing properly.

Monitors were more likely to fail the accuracy test because of overestimating BP rather than underestimating it; this suggests that underdiagnosis/treatment is less likely than overdiagnosis, which is reassuring.

This study suggests that the majority of monitors in current use by patients in UK primary care are likely to be accurate, and GPs should recommend that patients who are considering self-monitoring consult online lists of validated monitors (for example, https://bihsoc.org/bp-monitors/), replace monitors every 4 to 5 years, and avoid wrist models. Practices using such a policy could be confident that managing hypertension with such equipment is likely to be appropriate; other work by the authors suggests this will lead to better BP control.^5^
